# Policies and practices of climate change education in South Asia: towards a support framework for an impactful climate change adaptation

**DOI:** 10.1007/s44168-022-00028-z

**Published:** 2022-12-19

**Authors:** Marcellus F. Mbah, Ayesha Shingruf, Petra Molthan-Hill

**Affiliations:** 1 Manchester Institute of Education, School of Environment, Education and Development, University of Manchester, Oxford Road, Manchester, M13 9PL UK; 2 Nottingham Institute of Education (NIE), School of Social Sciences, Nottingham Trent University, Clifton Lane, Nottingham, NG11 8NS UK; 3 Sustainable Management and Education for Sustainable Development, Nottingham Business School, Nottingham Trent University, Nottingham, NG1 4FQ UK

**Keywords:** South Asia, Climate change education, Climate policies, Climate change adaptation, SDG13, Climate-change policy

## Abstract

South Asia is one of the most vulnerable regions to the impacts of climate change. While it has contributed the least to global greenhouse gas emissions, the people in the region face imminent threats to their health and well-being due to extreme weather events and a range of other climate-induced challenges, such as biodiversity loss and monsoon floods. Climate change education (CCE), if properly implemented, can prove to be vital in addressing such challenges and in helping people make informed decisions on adaptation strategies. To this end, this paper explores how CCE is addressed in the climate policies of South Asian countries. The paper also explores the implementation and operationalization of these policies within the respective countries. A content analysis of the climate policies of member states was conducted for the period 2007–2021. The findings revealed that climate change education has been highlighted within individual policies pertaining to formal and informal education, research and development, and public awareness initiatives. However, a gap analysis between policies and practices reveals that significant work is still needed for the effective implementation of policies supporting CCE at local levels. The current study seeks to contextualize these gaps by situating them within the relevant theoretical backdrops. In doing so, the research aims to make a meaningful contribution to the understanding of how CCE can be effectively deployed and delivered towards an impactful climate change adaptation, building on a support framework.

**Graphical Abstract:**

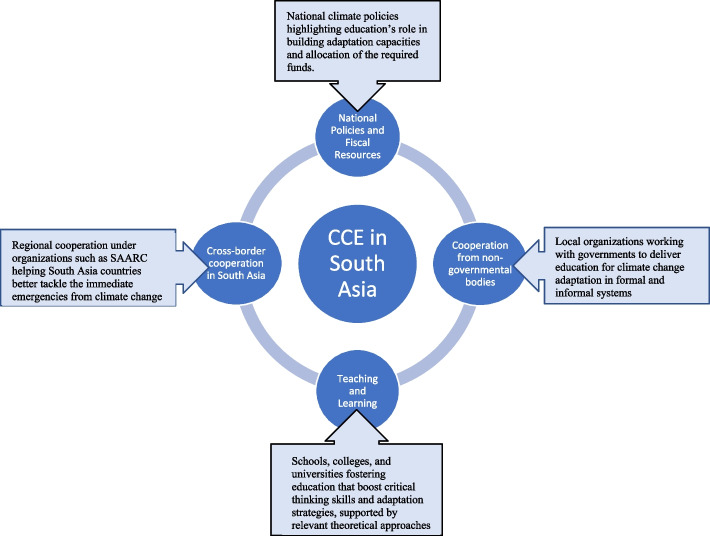

## Introduction

Human activities such as the burning of fossil fuels and deforestation have contributed significantly to the warming of our planet. Greenhouse gases (GHG) from human activities have been responsible for a 1.1°C rise in global temperatures since 1850 (IPCC, 2021). Furthermore, global average temperatures are projected to climb to 1.5°C or above within the next 20 years (IPCC, 2021). For our planet, a 1.5°C rise in temperature would exacerbate an already imbalanced ecosystem, resulting in extreme weather events that contribute to environmental damage, biodiversity loss, food insecurity, and the mass displacement of people (Heshmati, 2021). The effects of climate change may result in drastic ecological consequences that cascade down to affect all aspects of life on earth. The predicted increase in frequency and intensity of extreme weather events will have devastating consequences on communities around the world (IPCC, 2021; Shaw et al. 2022). This will be severely felt in South Asia, where a unique mix of socio-economic, political, and geographical factors make the region particularly vulnerable. Ironically, while South Asian countries have contributed the least GHG emissions globally, they are nevertheless among the countries worst affected by climate change (Aryal et al., 2020).

**Fig. 1:**
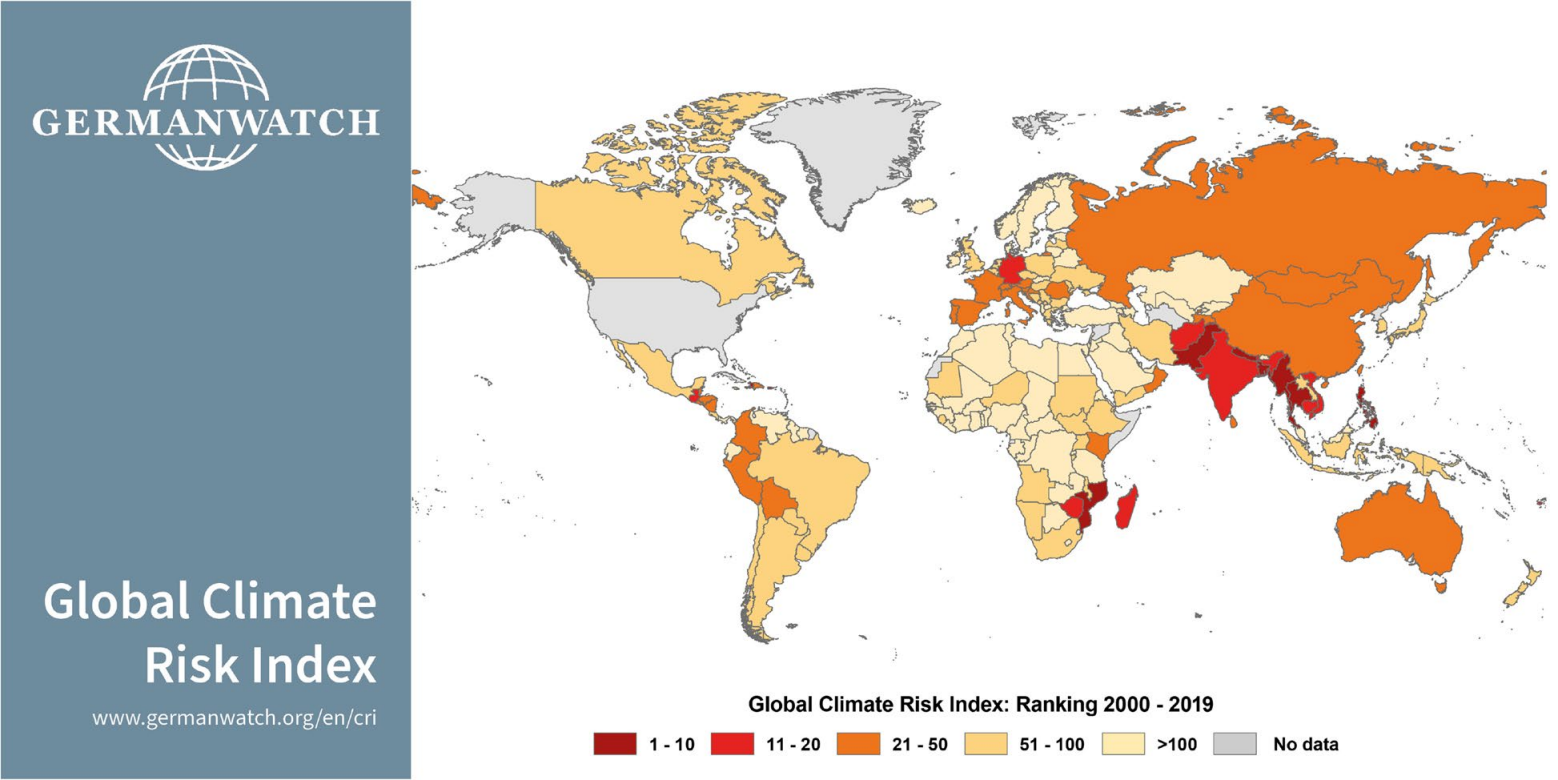
Global Climate Risk Index 2021: country rankings (2000–2019) depicting the level of vulnerability of South Asia and other regions to the adverse effects of climate change. Source: Eckstein, Künzel & Schäfer (2021), p. 15; Germanwatch and Munich Re NatCatSERVICE. Accessed 07 Oct. 2022

The impact of climate change will likely worsen the challenges already being faced by South Asia, including those related to hunger, poverty, security, inequality, and health (Zhenmin & Espinosa, 2019; Cramer et al., 2018; Hallegatte & Rozenberg, 2017). The Global Climate Risk Index 2021 (see Fig. 1) depicts that countries in this region face a greater risk of exposure and vulnerability to the adverse effects of climate change as compared to several other countries around the world. This justifies our focus on the region, and immediate and urgent action is needed to respond to this threat.

One of the responses to tackle the adverse impacts of climate change can be found within the 2030 Agenda for Sustainable Development. The implementation of the 17 interlinked global Sustainable Development Goals (SDGs), which make up the 2030 Agenda, will help move our planet to a more sustainable and fairer future (United Nations, 2015). Of more relevance to this paper is goal 13 (SDG13), which addresses urgent action to combat climate change, highlighting the need to “improve education, awareness-raising and human and institutional capacity on climate change mitigation, adaptation, impact reduction and early warning” in its target 13.3 (United Nations, 2015). Education as a tool to combat climate change has also previously been stressed in Article 6 of the United Nations Framework Convention on Climate Change (UNFCCC) and was later restated in Article 12 of the Paris Agreement, wherein measures to enhance climate change education and public awareness were emphasized (UNFCCC 2015, 10). As we highlight climate change education in this paper, it is about learning to understand, engage, and confront the adverse effects of climate change and this is not limited to formal or informal educational architectures but captures both, as would be evidenced in the different sections of this paper.

Knowing that climate change is a threat multiplier, collaborative global actions are required, rooted in problem-solving, and attention must be drawn to climate change education (CCE) and its development for effective adaptation practices in South Asia. Given how severely South Asian countries are affected by the impacts of climate change (Sen et al., 2017; Liu et al., 2017; Hasnat et al., 2018; Schafer, 2019), the focus of CCE on adaptation is significant. Here, we defined climate change adaptation as mechanisms or methodologies put in place to respond promptly and adequately or effectively to reduce the vulnerability of environmental and human systems to the impacts of climate change (Molthan-Hill et al., 2022). It should be noted that as the degree of the impact of climate change varies across nations and regions, if one wants to anchor education activities locally to facilitate adaptation processes, place-based specific conditions can play a key role (Ajaps and Mbah, 2022).

The significance of CCE cannot be understated. It can play a beneficial role in enabling communities to make informed decisions when faced with the threats of climate change (UNESCO, 2017). One of the major benefits of CCE is the “multiplier effect” which has been discussed by Mochizuki and Bryan (2015). According to this, individuals acquire knowledge and share it by passing it on to their communities. This offers a sustainable source of local knowledge which can be carried forward (Mochizuki and Bryan, 2015). Furthermore, CCE offers communities a chance to gain new knowledge while simultaneously helping them build adaptive strategies needed to manage climate-induced challenges. This can be achieved through formal and informal education, as well as awareness programs that can inform communities about climate change mitigation and adaptation techniques (Reid, 2019). Crucially, CCE can inspire learners and communities to positively change their attitudes toward climate action or consider relevant climate change adaptation strategies. In addition to addressing knowledge deficits, CCE can help foster an atmosphere of social cohesion and shared responsibility. Attitudinal and behavioral changes accruing from CCE have the potential to shift the weight of public opinion as new social norms become established.

Therefore, CCE is a valuable tool, and its integration in the national policies of South Asia has the potential to greatly foster the response to climate change. It is through education that young people and communities can be mobilized and offered the knowledge and skillset necessary to adapt to climate-related challenges. The impending danger posed by the climate crisis calls for an analysis of the existing national policies and practices of South Asian countries as they relate to CCE. Through this, we may arrive at possible solutions for climate change that align with a more sustainable and safer future. The research, therefore, aims to make a meaningful contribution to the understanding of how CCE can be effectively deployed and delivered towards an impactful climate change adaptation, building on a support framework. To achieve this aim, the following research questions will be addressed: (a) what are the existing policies in South Asia that support education for climate change adaptation in the region, (b) what are the corresponding practices that align with existing policies on education for climate change adaptation in the region, (c) what are the gaps between policies and practices that support education for climate change adaptation, and (d) what framework can be conceived that would enable the narrowing of the gaps identified.

## Background

South Asia is a region that consists of the countries of Afghanistan, Bangladesh, Bhutan, India, Maldives, Nepal, Pakistan, and Sri Lanka. As of 2020, the region has an overwhelming population of 1.85 billion (The World Bank, 2020). It is home to an incredibly diverse landscape ranging from the Himalayan peaks to fertile lands, forests, and coastal cities. Because of its many unique characteristics and geographical location, South Asia remains especially prone to natural disasters or climate-induced challenges. The Working Group II Contribution to the Sixth Assessment Report of the Intergovernmental Panel on Climate Change (IPCC) (Shaw et al. 2022) may be a testament to the challenges South Asian communities are expected to face in the next few years, including rising temperatures which are likely to increase the likelihood of the threat of long spell of heatwaves across Asia, leading to droughts in arid and semiarid areas, delays and weakening of the monsoon circulation, floods in monsoon regions, and glacier melting. The impacts of climate change in this region are already being felt across countries. The next section will explore some of these climate-related challenges that touch on food availability, water resources, health and well-being, and extreme weather events before moving on to an analysis of national policies as they relate to CCE in South Asia.

### Food availability

South Asia depends heavily on agriculture, and farmers in this region alone have been feeding over 20% of the global population (Rasul, 2021). Drawing on the Working Group II Contribution to the Sixth Assessment Report of the Intergovernmental Panel on Climate Change (IPCC) (Shaw et al. 2022), it is projected that in the continent, as well as the region under consideration, agriculture and food security will be affected considerably, particularly in the area of cereal production by the end of the current century, leading to malnutrition among the poor and socially disadvantaged sections of the population due to climate change.

Apart from cereal production, recent studies have determined that climate change would also impact the production of major crops such as maize, rice, wheat, and soybean. A previous analysis from India supports this estimation as it found that global warming was responsible for a 5.2% decrease in wheat yield between the years 1981 and 2009 (Gupta et al., 2017). With the current rates of global warming, it is estimated that Bangladesh and India will see the highest percentage of decline in wheat production (60%), followed by Pakistan (27%). Rice production is also expected to decline significantly in India (40%) and Nepal (32%) (Rasul, 2021). For South Asian countries, this will not only impact crop availability but will have severe consequences on market prices, affecting millions of people in the region (Rasul, 2021; Aryal et al., 2020). This is because food scarcity can drive demand, and demand can drive higher prices, and affordability can only be achieved by those with high incomes or earnings. Additionally, climate change in South Asia will have staggering economic consequences with the region expected to lose more than 1.8% of its annual gross domestic product (GDP) by 2050 (Ahmed and Suphachalasai, 2014).

### Water resources

Some parts of Afghanistan, India, and Pakistan are dependent on glacial melts, which provide water for the cultivation of crops in high altitudes. Local studies conducted in the mountainous regions of the Himalayas have confirmed that water shortages are already being experienced in such communities and are also affecting food security (Rasul and Molden, 2019). In India, Bangladesh, and Sri Lanka, groundwater tables are decreasing. Elsewhere, Maldives is facing a threat to freshwater resources with an increase in the frequency of dry weather (Hasnat et al., 2018; Schafer, 2019). With the melting glaciers and prolonged droughts in South Asia, water stress conditions have become a multi-faceted phenomenon that will not only bring damage to the communities living downstream but will also reduce the water supply for people living in other parts of the region (IPCC, 2021; Shaw et al. 2022).

### Health and well-being

Brooks et al. (2019) established the link between disease and climate change when they studied the rise of transmission of vector-borne diseases corresponding to a temperature rise. This is consistent with findings in the IPCC Working Group II Contribution to the Sixth Assessment Report, which ascertains a co-relation between vector-borne and water-borne diseases, mental disorders, allergy-related illnesses, undernutrition, and climate-induced hazards such as flooding, heatwaves, and drought, among others (Shaw et al. 2022). In a recent study, the increased incidence of malaria in Bhutan has also been associated with an increase in rainfall and temperatures (Wangdi et al., 2020). Furthermore, the threat of water-borne diseases is aggravated by climate variabilities in temperature, rainfall, and humidity (Liu et al., 2017). As South Asia is prone to floods, water-borne diseases such as cholera will also likely increase (Sen et al., 2017). This poses additional threats to vulnerable population groups such as young children living in an already vulnerable region. Hence, it is crucial to develop and implement adaptation practices that cater for the health and well-being of people living in South Asia.

### Extreme weather events

**Fig. 2:**
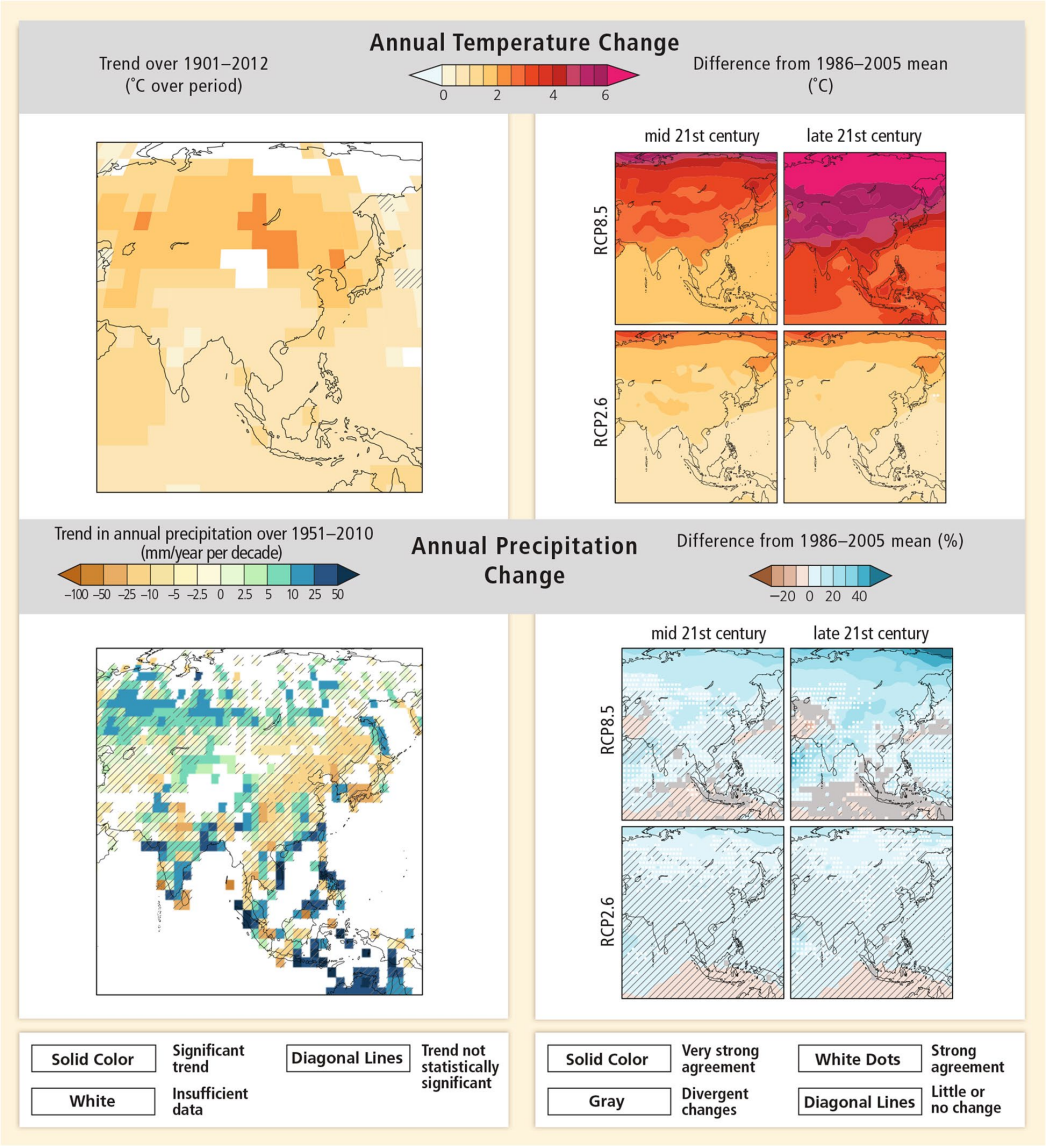
Annual temperature and precipitation changes in South Asia as a result of climate change. Source: IPCC (2021) Working Group I Contribution to the Sixth Assessment Report, accessed 10 March 2022

The countries in South Asia are currently experiencing rising sea levels coupled with an imminent risk of melting glaciers as temperatures around the world continue to rise (IPCC, 2021). The Working Group I Contribution to the Sixth Assessment Report of the Intergovernmental Panel on Climate Change captured an analysis of weather patterns in South Asia, showing that in recent years the region has seen a rise in annual temperatures (see Fig. 2). Heatwaves and humid heat stress in these countries are expected to worsen in the coming years, and monsoon activity is likely to become more intense (Shaw et al., 2022). This is already resulting in floods, as is evident in India, where more than 18 million people were affected as an aftermath of the 2021 floods (Balmain, 2021). Similarly, Bangladesh saw more than 200,000 people affected by floods in Cox’s Bazar, and Nepal saw destructive floods swept away homes and communities (Paul, 2021; Ellis-Petersen, 2021). Living in South Asia is proving to be a challenge as more destruction is expected, with the Himalayan mountains losing snow volume and glaciers in the region experiencing their own volume decline (IPCC, 2021). Immediate interventions are now required to attenuate the predictable and damaging effects of climate change.

This section has revealed some of the major challenges being faced by South Asian countries if immediate steps are not taken to tackle climate change. Previously, this paper discussed the scope of CCE in offering effective strategies for climate action, particularly highlighting adaptation. Attention must now be drawn to a relevant approach to CCE as a framework that can support the provision of sustainable solutions that would aid communities in moving forward in the face of the adverse effects of climate change. To further address this, there is a need to examine some theoretical propositions.

#### Theoretical underpinning

Given the threats posed by climate change, it is essential to turn to the opportunities a sustainable educational framework can create in countering them. CCE can advance ideas for consideration in South Asian communities that would instigate relevant actions or behavioral patterns in an informed manner. Several scholars have already accentuated education, with different underlining theories/concepts, as a valuable tool for climate change adaptation (Molthan-Hill et al., 2022; Apollo and Mbah, 2021; Mbah et al., 2021; Feinstein and Mach, 2020; Krasny and DuBois, 2019; Wamsler et al., 2012). Additionally, the pedagogical approach captured in the Education for Sustainable Development (ESD) 2030 roadmap highlights the need for educators to “employ interactive, project-based, learner-centred pedagogy,” as well as provide a learning context which would “enable learners to live what they learn and learn what they live” (UNESCO, 2020, p. 8). The theories discussed in this section such as the theory of change (Jackson, 2013; Connell and Kubisch, 1998), critical pedagogy (Giroux, 2020; McLaren, 2015; Mathews, 2014; Freire, 1968), and the place attachment theory (Quinn et al., 2015) support the ESD (2030) roadmap and have been chosen for their relevance in the design and implementation of a useful framework that can buttress an impactful education for climate change adaptation as evidenced in the extant literature. The ultimate goal here is to build the capacity of communities to successfully live with what they learn in the context of challenging circumstances of climate-induced challenges.

### Theory of change

Theory of change (ToC) is an approach used in international development which lays out a roadmap to achieve a long-term goal (Stein and Valter, 2012). This approach imagines a “big picture” outcome and considers the steps needed to achieve its main goal (Bours et al., 2014). This is well-suited for multi-faceted phenomena such as climate change adaptation because it offers a lens for contextual analysis. The theory has the propensity to facilitate learning through a concrete embodiment of long-term best practices. Given its tested and proven potential to create a desirable impact, the DFID’s guidelines provide a broader perspective, underscoring the need for implementers of the ToC to draw on objectives and frameworks that are owned nationally, supported with evidence that guarantees behavioral changes and economic transformation among others (Stein and Valter, 2012).

Consequently, and through ToC, a CCE program can be conceived that can benefit local communities in South Asia as it will focus on achieving a positive outcome for the region. Changes brought about via the introduction of CCE can be evaluated based on the community’s location, sector, or social group. While it may be time-consuming to develop a program using ToC as the primary approach, its flexibility creates space for reflection and learning which can be an asset for the development of an efficient adaptation program (Pringle & Thomas, 2019).

### Place attachment theory

CCE can also be viewed to include a sense of “place” and “identity” between the individuals and the locations they are connected with. This is supported by the place attachment theory, whereby people associate a positive attachment with a community and place (Altman and Low 1992; Brown and Perkins, 1992; Fried 2000; Ajaps and Mbah, 2022). When we recognize that attachment to a place is holistic and is continuously changing, we can identify its influence on relevant challenges and adaptation practices (Quinn et al., 2015). Bronen and Chapin (2013) conducted a study in Alaska in which they concluded that a high sense of identity and belonging made people more willing to take collective action against the impacts of climate change. As a regional identity of South Asia already exists, place attachment theory can be effectively incorporated into CCE to encourage the embedding of sustainable and impactful solutions to the need for effective climate change adaptation strategies that are relatable.

### Critical pedagogy

Oberman and Sainz (2021) stated that along with addressing the scientific knowledge behind climate change, CCE must also capture appropriate responses. Additionally, Zeidler and Nichols (2009) argue that critical thinking among learners needs to be encouraged so that they may be able to make informed judgments by critically analyzing, evaluating, and interpreting the problems they face due to climate change. This notion of critical thinking requires students to critique power structures and understand how climate change impacts people from different social groups (Lim, 2015). This aligns with Freire’s idea of critical pedagogy and the critique of education as a system that reinforces power structures and inequalities (Friere, 2018). Freire (1968) made reference to the “banking concept of education” to explain how such power structures are maintained. According to this concept, teachers “deposit” information into the cognitive apparatus of students, which they learn in incurious ways. CCE programs must avoid such a top-down transmission of knowledge to allow both educators and learners to benefit from mutual engagement and knowledge co-construction. The delivery of education for impactful climate change adaptation that captures critical thinking skills must be one that views students as individuals capable of holistic understanding and transformation of their realities rather than as individuals who are mere recipients of knowledge imparted upon them (Freire, 1968). In this light, CCE, with the accompanying desirable effects, must view students as agents of change, capable of critically evaluating the challenges faced by their communities in order to support climate change adaptation.

### Critical consciousness and praxis

Freire’s idea of critical consciousness requires that students examine and reflect on the challenges in their society and take action to reform their experiences (Freire, 2005). Only through such participation with their own realities will they be able to transform the world around them. The relationship between the reflection, which students participate in, captured in their lived experiences, and the actions they conclude from it, is what Freire terms as *praxis* (Freire, 2005). Through this, students will be encouraged to contribute toward climate action and explore ways of reshaping their futures (Kirylo et al., 2010). Therefore, these ideas of critical consciousness and praxis can be readily employed within CCE to enable students to make the most of the scheme, heighten the connection between policy and practice, and analyze their experiences so that they may achieve a future that aligns with SDG13 and a sustainable environment.

Given these theoretical insights, the nature of impactful adaptation strategies resulting from climate change education also requires a holistic approach embedded in system thinking (Roychoudhury et al. 2017; Shepardson et al., 2012), whereby the complexities that underpin climate change and its adverse effects and relationships can be ascertained. Through the architecture of system thinking, learners can grasp very valuable insights into climate variations, causes, and effects and take appropriate measures to implement sustainable adaptation schemes. At the nexus of these theoretical frameworks, this paper argues that learners can be more adequately equipped with climate change adaptation knowledge if they can question, critique, engage in system thinking, and assess their experiences. Moreover, the implementation of national policies on CCE must be devised in a manner that teaches young people and communities to properly interrogate and apply adaptation strategies that are sustainable in their local contexts. Educators also have an obligation to create an enabling environment whereby learners can employ local knowledge and propose actions for climate change adaptation. Using the theories mentioned in this section as a foundation, a holistic framework for an impactful education on climate change adaptation can be envisaged and implemented in South Asian countries.

## Methodology

In addressing the research questions, we seek to analyze CCE policies and practices in South Asia and to offer a support framework that aligns with SDG13 in order to meet the goals necessary for adaptation. For this reason, we reviewed national policies and other relevant gray literature on climate change in the eight countries that make up the target region by using a qualitative research design. Considering a conventional quantitative approach for this study would have overlooked the intention of inductively constructing meanings from valuable texts or applicable policy documents. However, there are some limitations to qualitative research that touches on the fact that they are not easily replicable and that traditional concepts of validity and reliability are challenging to be applied (Langdridge & Hagger-Johnson, 2009). Nonetheless, as argued by Mbah (2016), a qualitative research approach has several advantages in that it “recognises the subjective elements of the research process; it is not limited to one perspective on different social subjects and often generates unexpected insights” p.1232.

Through government databases and reliable websites, national climate policies of individual countries were obtained for the analysis. The policies obtained were realized between 2007 and 2021. They were reviewed to capture any updates or developments by ensuring that the data collected was current and relevant to this research. Furthermore, an extensive review of relevant gray literature on target countries was conducted to understand how CCE is being practiced across South Asian communities. It is possible that a few existing policies and practices of CCE in the region may have been missed in this study as a result of a lack of access or no documentation. Furthermore, as South Asia is a region that is experiencing extreme poverty and inequality, the implementation of effective climate policies may not have been a priority in some countries. Notwithstanding, the documents accessed and reviewed provide significant insights into current gaps between the policies and practices of CCE. The potential for further development of key policies to respond to the impacts of climate change in practical ways that are feasible is further discussed.

### Data analysis

Adopting the right approach to analyzing a qualitative dataset would ensure the reliability of the findings. For this study, a content analysis of national policies and practices on climate change in South Asia can be justified. Content analysis is helpful for qualitative research in studying trends and patterns. Furthermore, it helps in analyzing any visible co-relations between the data gathered at different points in time (Stemler, 2001). Content analysis also makes for an efficient approach as large quantities of data are analyzed systematically (GAO, 1996). Previously, content analysis has been used in studies where existing data is limited as it allows for new insights and categories to develop (Kondracki & Wellman, 2002). This is relevant to the context of this paper as CCE in South Asia has not been widely studied, and there is limited information on the subject. Reliability was further enhanced by ensuring individual national policies were read and reread various times to identify key terms which emerged frequently, and an organized set of themes were established. During this process, specific terms were highlighted in the data and arranged based on their association with CCE and SDG13 such as “education,” “sustainability,” “climate change,” “schools,” “public awareness,” “universities,” and “research.” In the context of the analysis, we explored how CCE has been captured in policy documents such as national curriculums and whether there are corresponding or associated practical elements.

### Findings

The national climate change policies of countries in South Asia studied are summarized in Table 1. This section drew on a content analysis to break down the key references to CCE. The overarching aims, targets, or approaches to CCE in South Asian countries are identified as themes and are discussed in detail further. These themes highlight the policies and approaches that exist to support CCE in the region and include climate change education in schools, climate change research and development, and public awareness interventions.

**Table 1:** CCE policies in South Asia

Country	Climate change policy	Overarching references to CCE	Main targets for CCE practices	Links
Afghanistan	Disaster Management Strategy, 2014 National Environment Strategy (NES), 2007	Environmental education and building capacity and resilience at a local level	• Institutional mainstreaming of environmental issues through development programs • Curriculum designed to train rural communities on capacity building	Govt. of Afghanistan, 2014 UNFCCC, 2009
Bangladesh	National Plan for Disaster Management, 2017 Climate Change Strategy and Action Plan, 2008	Research and development, capacity building, and institutional strengthening	• Incorporating disaster risk knowledge in formal and informal education • Supporting and promoting disaster risk research in academics	Govt. of Bangladesh, 2017 Govt. of Bangladesh, 2008
Bhutan	National Environment Strategy, 2020 Climate Change Policy of the Kingdom of Bhutan, 2020	Research, awareness, and education to support climate change adaptation, capacity building	• Curriculum includes environment and climate change for different levels of education • Supporting research and disseminating knowledge for informed decision-making on climate change	National Environment Commission, 2020 Kingdom of Bhutan, 2020
India	NDC, 2016 National Action Plan on Climate Change (NAPCC), 2009	Research and communication-based actions Knowledge systems that engage research and development on climate science	• Creation of knowledge systems that support research • Developing human resource for adaptation by reforming school and college curricula	UNFCCC, 2016 Govt. of India, 2009
Maldives	Maldives Climate Change Policy Framework (MCCPF), 2015	Research, capacity building, and institutional strengthening Promoting sustainable practices through awareness, education, and training	• Mobilizing youth to engage in climate change understanding • Implement knowledge within secondary and tertiary schools, as well as vocational education • Encourage research on climate change issues	UNISDR, 2021
Nepal	National Climate Change Policy, 2020	Involves governmental and non-governmental organizations to promote academic research and sustainable practices for the local communities	• Knowledge on climate change adaptation to be incorporated in formal and non-formal curricula • Farmers’ schools to guide farmers on sustainable crop production	Govt. of Nepal, 2020
Pakistan	National Climate Change Policy (NCCP), 2021 Pakistan’s 2025 Vision, 2014	Promotes sustainable adaptation, capacity building, and institutional strengthening	• CCE in secondary and tertiary schools by 2030 • Develop climate change adaptation planning within formal education systems at all levels • Public awareness on sustainable management practices	Govt. of Pakistan, 2021 Govt. of Pakistan, 2014
Sri Lanka	National Action Plan for Haritha Lanka Programme, 2009 Climate Change Policy, 2012	Promotes climate education and sustainable development knowledge, awareness, capacity building, research, and development	• To integrate CCE and sustainable development in school and universities • Promote behavioral changes in schools and encourage climate change research at academic levels	Govt. of Sri Lanka, 2019 Govt. of Sri Lanka, 2012

Climate policies in South Asia are prevalent with references to research and development and public awareness on adaptation strategies. Additionally, some countries are looking to include CCE into formal educational institutes to strengthen capacity and resilience against climate change impacts. There are therefore possibilities to mainstream CCE in South Asia educational systems as it aligns with SDG13 and target 3.

#### Climate change education in schools

In most countries in South Asia, education has been mentioned as an important tool to aid adaptation to climate change. For example, Pakistan’s formal education systems are expected to include CCE across all levels, with a special emphasis on higher education. It has also pledged under its Nationally Determined Contributions (NDCs) to include CCE in the curriculum of secondary and tertiary education systems by 2030 (Government of Pakistan, 2021). In Afghanistan, education on climate change is listed as one of the country’s key approaches to disaster prevention (Government of Islamic Republic of Afghanistan, 2011). However, the policy does not detail the delivery and recipients of such a program. While the policy itself may have the potential to be improved, the future of CCE in Afghanistan is unpredictable. It must be taken into consideration that at the time of writing this paper Afghanistan is under the Taliban and any development in policies and practices will need to be investigated in light of recent political events in the country (Islam et al., 2022).

Elsewhere in Sri Lanka, emphasis is placed on learning to address the knowledge gap on issues of climate change within the formal education system (National Adaptation Plan for Climate Change, 2016). More specifically, Sri Lanka aims to integrate physical, ecological, and environmentally friendly policies and practices as part of their school and university curricula (National Action Plan for Haritha Lanka, 2009). Additionally, Sri Lanka’s policy on sustainable consumption and production (2019) has targeted sustainable knowledge and practices to be introduced as a life skill in schools, universities, and vocational education systems by 2025. Bhutan’s ministry of education is developing a curriculum on environment and climate change and incorporating knowledge on climate change issues at different levels of the education systems (National Environment Commission, 2020). Bhutan also acknowledges that knowledge about the environment can be gained via schooling; thus, environmental education has been mainstreamed in schools from pre-primary to higher secondary levels. Maldives on the other hand focuses on the mobilization of the youth by engaging them through programs that embed information on climate change adaptation in the secondary and tertiary schools, as well as vocational education (UNISDR, 2021). Nepal’s updated National Climate Change Policy (2019) is integrating climate knowledge within both formal and non-formal education sectors and hopes to carry out adaptation programs in educational institutions.

#### Climate change research and development

India’s National Action Plan on Climate Change (NAPCC) was announced in 2009 and includes eight targets for adaptation and mitigation. One of these targets is to promote knowledge on climate change by implementing a research and action-based system (Government of India, 2008a). In its Nationally Determined Contribution (NDC), India recognizes the importance of trained professionals to tackle the challenges of climate change. In Bangladesh, the climate change policy includes action points for disaster management, infrastructure, and research and knowledge management. Within this policy context, research and knowledge management are intended to predict the likely scale and timing of climate change impacts on different sectors. Similarly, Sri Lanka’s government has stated that it is on a mission to develop a generation which is sensitive to the impacts of climate change. It intends to do so by promoting research and development on climate change issues at a national level. Within its policy goals, Sri Lanka has an aim to educate and empower both local governments and academia to understand and implement adaptation practices in vulnerable areas of the country.

#### Public awareness

In Pakistan, the National Climate Change Policy (2021) acknowledges the need to raise awareness of climate change on a much greater scale than is currently being carried out in the country. For this, it aims to develop a climate change awareness program which will involve communities and guide them on sustainable management practices for forests, water, and energy. Similarly in India, importance is placed upon public awareness within different sectors, including within its National Mission for Sustaining the Himalayan Ecosystem (Government of India, 2008b), which aims to develop the knowledge around climate change adaptation for its communities. Within their policy framework, Bangladesh and Sri Lanka also focus on public awareness to develop climate resilience among communities. Nepal and Maldives both have climate change policies, which reflect their commitment to sustainable development practices as they seek to empower local communities to build climate resilience and make efficient use of their resources with principles focused on fairness. The climate change policy of Bhutan has targeted resilience and capacity building, as well as enhancing climate knowledge to better implement adaptation strategies by supporting institutions in data collection and disseminating information at a local level.

While these policies are mentioned in government documents at the national level, attention must also be drawn to the practices being carried out in communities and whether they reflect the commitments pledged to combat climate change. Table 2 highlights the summary of CCE practices in South Asia, and in the ensuing section, the evidences in the targeted countries are examined in depth. The focal points include governments’ focus on increasing climate literacy to tackle climate change, and public education to create an awareness on environmental issues, disaster management, and conservation practices.

**Table 2:** CCE practices in South Asia

Country	Key evidence of CCE	Source
Afghanistan	Conservation Organization of Afghan Mountains (COAM) aimed to raise awareness among local communities on conservative practices	UNEP, Afghanistan
Bangladesh	School curriculum contains some aspects of disaster management and environment, but full-scale CCE implementation has not yet been developed	CCE and sustainable education in Bangladesh
Bhutan	Royal Society for Protection and Nature focuses environmental education on conservation and is delivered in all formal and non-formal educational institutes	Royal Society for Protection and Nature, Bhutan
India	Climate change programs focus on climate literacy, and training is delivered in schools, universities, and vocational institutes	Centre for Environmental Education
Nepal	Climate change knowledge management centers, and farmer schools	Nepal’s education and management centers
Maldives	NGOs in Maldives promote climate adaptation strategies among young students and local communities	Bluepeace Live & Learn, Maldives
Pakistan	Action-based learning of climate literacy in schools and promotion of environmentally friendly practices among young students	Clean Green School Program, Pakistan
Sri Lanka	Open School Program runs across the country and supports rural education on environmentally friendly practices such as reducing carbon emissions	UNESCO, Sri Lanka

#### Practices of CCE

Governments in South Asia are increasingly addressing climate change in their countries. To adapt to climate-related events, countries in this region are educating and training students, as well as local communities. There are various organizations and projects in place which are focused on CCE in South Asia and while the list does not capture every initiative, the most influential ones have been identified in Table 2 and discussed below.

#### Climate literacy

By climate literacy, reference is made here to the acquisition of basic information that is deemed significant for communities and their inhabitants to grasp an understanding of the Earth’s climate, consequences of human actions on climate change, and approaches to mitigation, but most importantly adaptation. India has set up its Centre for Environment Education (CEE) under its Ministry of Environment, Forest and Climate Change. One of the main goals of this center is to develop and promote educational material as it relates to climate change in schools, universities, and other training programs (Sarabhai & Joshi, 2018). It works with both formal and informal education sectors to enhance knowledge on issues of climate change, adaptation strategies, and biodiversity conservation by delivering interactive activities and programs.

Similarly, Pakistan’s Clean Green School Program under the partnership with the country’s ministry of climate change is involved in imparting action-based learning in schools on climate literacy (WaterAid, 2019; Jamal, 2019). Under this program, students in schools and colleges are gaining knowledge on issues of climate change, and ways to reduce disaster risks, and engage in environmentally friendly practices. Currently, the program runs only in schools and colleges of the country’s capital city which restricts climate literacy and knowledge on adaptation from being delivered to young students across the country. On the other hand, the Nepal Climate Change Knowledge Management Center has been set up under Nepal’s National Adaptation Program of Action (NAPA) which strengthens capacity building by delivering workshops, training, and other interactive programs in schools. By engaging young students through media and art, the management center sensitizes young students in rural areas across Nepal on climate change adaptation practices (Climate & Development Knowledge Network, 2013).

#### Public education

South Asian countries are also integrating CCE outside of schools and colleges by raising public awareness of climate issues among local communities. This is true for Nepal where specialized famers’ schools have been established and farmers are able to gain knowledge on crop development using indigenous knowledge systems (Jha et al., 2020). In Maldives, non-governmental organizations are working to improve awareness on environmental protection through training, workshops, and interactive sessions in local communities. A good example of public education on climate change is in Sri Lanka where under the Open School Program rural communities are taught on how to adopt best practices for the environment. These include educating farmer communities on organic farming, managing waste and delivering education on green practices (UNESCO, 2018).

#### Education on conservation practices and disaster management

A recent study has shown that in Bhutan there is an emphasis placed on environmental education and protection in school curricula (Jeronen et al., 2022). Using the principles of conservation and transformation, Bhutan is reviewing and improving its environmental education curriculum. Additionally, the country’s Royal Society for Protection of Nature is engaging with students, monastic institutions, non-formal education, and local communities to encourage and support conservation practices. Afghanistan’s NGO Conservation Organization of Afghan Mountain in collaboration with UNEP supported environmental awareness programs in remote areas of Afghanistan; however, data on its progress and development is currently limited and outdated (UNEP, 2017). In Bangladesh, school curricula include imparting knowledge on climate-related disasters, their causes, and preventions. However, there is an absence of a holistic approach to CCE in primary, secondary, and higher education. Consequently, it can be ascertained the country lacks the key knowledge on adaptation to climate change (Shohel et al., 2021).

These practices are an important step ahead for South Asia, but there are prominent gaps that exist in the effective and inclusive implementation of CCE in the region that would provide a sustainable approach to adaptation to climate change. The next section highlights some underlining reasons for these gaps.

### Underlining reasons for the gaps between policies and practices

Gogoi and others (2017) discuss that most of the policies on climate change adaptation are not “implementation-ready.” Moreover, as there is no place for accountability within these climate policies, many governments do not prioritize their development. Due to a lack of finances, agreements, and incentives, climate change adaptation policies are not developed for further action (Gogoi et al., 2017). It has also been argued that climate policies tend to be aid-driven and top-down in their approach (Ojha et al., 2016). Thus, this impacts a context-based implementation of CCE that caters to the needs of local communities. Zafarullah and Huque (2018) argue that environmental governance in South Asia has generally been state-driven, which disallows different stakeholders from taking part in the policy process. Because of the lack of attention to the diverse challenges faced by local communities, climate change adaptation in South Asia offers insufficient approaches to the disaster risks involved.

Another report published by UNICEF (2021a) highlights the gap between policies and practices more accurately in South Asia. The Youth Perspectives on Climate Change and Education in South Asia measured the experiences of young people in South Asia’s eight countries, and their perceptions of and involvement in climate change learning and action opportunities within their schools and communities. Through this report, it was found that most young people in South Asia were not able to explain climate change or global warming. Generally, the respondents who had learned about climate change in schools reported that they had learned through Geography or Science subjects. Furthermore, 69% of the young people in the study reported feeling “little worried” or “very worried” about the impacts of climate change in the future. In Sri Lanka alone, 59% of the respondents said they were “extremely worried,” painting a grim picture. When asked what they most wanted to learn about climate change, many of the respondents in the study showed interest to learn about “all” aspects of climate change, which included local action, prevention, causes, impacts, and others.

This report shows the crucial role education and schools can play in helping young people not only understand the causes and issues of climate change, but also in guiding them to take appropriate climate-oriented actions. To achieve this, a multi-disciplinary approach to CCE must be realized in formal and informal education.

It is essential that a comprehensive and inclusive framework is established to seek the desired results on adaptation. Despite the gaps between policies and practices and their underlining reasons, the analysis has shown that there are institutions in South Asia that exercise the potential to support a useful CCE framework. In the following section, a discussion is presented aimed at understanding where possibilities for an impactful CCE in South Asia can emerge, as well as ways in which a support framework can be established to meet the needs for climate change adaptation in South Asian communities.

## Discussion

We have seen from the earlier sections of this paper that policies as they relate to CCE exist in the climate-related frameworks of South Asian countries; however, these policies remain underdeveloped. This is due to multiple reasons which include limited resources and finances on educational facilities and in some cases, lack of determination of governments to deliver on their targets (Asadullah et al., 2020). Therefore, a contextual and step-by-step approach to CCE in South Asia with adequate investment of resources is needed. Such a framework for CCE should be place relevant and aligned with the theory of change (ToC) and Freire’s ideas of critical pedagogy, conscientization, and praxis. For instance, the ToC’s focus on setting strategic plans to achieve desired outcomes will be beneficial towards the design and implementation of CCE in South Asia. There are opportunities within this practice to explore context-based solutions, which will be an asset for South Asia communities as there is a diversity of worldviews that exists in the region. Moreover, as students have an emotional attachment to the diverse landscapes of South Asia, the place attachment theory may also be a helpful lens through which policymakers can derive solutions to maintain an effective CCE program. With insights into critical pedagogy, students can critique, as well as take efficient actions for the improvement and sustainability of their environment.

As opposed to the “banking concept of education,” we must allow young students and local communities to engage and analyze their environmental challenges themselves. It is only through a mutual and respectful dialogue between the educator and the learner will an impactful action-oriented climate change education be imagined. Moreover, Freire’s idea of an appropriate pedagogy respected the use of a cultural context and placed crucial importance on the culture and indigenous knowledge students possess (Au and Apple, 2007). For CCE, this is a necessary starting point as local communities in South Asia are already aware of the challenges they are facing due to the changing climate. A CCE program which supports SDG13 must then incorporate the ideas of knowledge co-creation and transfer between the teacher and student, and power structures that reinforce epistemic violence and hegemony must be dismantled.

Furthermore, the key to strengthening CCE is by realizing the vision to create resilient communities that can adapt to the vulnerabilities posed by climate change. In this light, ToC can enable opportunities for policymakers and climate change adaptation practitioners to create a positive vision, describe the problem, and explore interventions and activities focused on building adaptation strategies. This can be done by reviewing past research and documents on climate change impacts and adaptation. Additionally, the backward mapping approach to identify outcomes will help in creating themes or key areas of focus within CCE. This will provide greater clarity when activities and programs are designed to achieve the outcomes set in place (Pringle and Thomas, 2019). These outcomes must be realized within localized contexts as climate change adaptation is often tailored to specific conditions. Therefore, incorporating ToC in CCE will not only help communities effectively respond to the climate crisis but will bring forth reflections and key insights from community engagements, which can be used in the intervention processes elsewhere. ToC’s framework possesses overlapping features with Freire’s idea on pedagogy in that both frameworks require and encourage open dialogue to strengthen shared values, which are necessary to develop climate adaptation programs (Bours et al., 2014).

The IPCC’s reports on climate change in South Asia reiterate the regional emergency and attention must now be paid to the essential steps needed to help communities adapt. Adaptation efforts can reduce and control the impacts of climate change, but they must be implemented immediately. As understood under SDG13, education on adaptation, impact reduction, and early warning can offer a sustainable framework in surviving with increasing global warming. While South Asian countries remain dedicated to meeting their SDG targets, they have generally lagged in providing quality education to young people with enrollment levels significantly less than the global average (ESCAP, 2021; Kumar et al., 2016). Moreover, gender and socio-economic factors also play a part towards access to education, especially tertiary education in South Asia which further hinders the progress for sustainable development. For climate change adaptation, this results in major setbacks as it slows down progress for youth mobilization, climate awareness, and knowledge sharing as targeted in the policies of South Asian countries. It also reduces the countries’ abilities to adapt to climate change fast and as successfully as possible.

South Asia has been experiencing low enrolment rates in schools. A report published by UNESCO (2014) that highlights the educational enrollment data gathered for the year 2014 indicates 7.57 million children between the ages of 5 to 10 were out of school in Bangladesh, India, Pakistan, and Sri Lanka. In the targeted countries where data was gathered, an additional 25.29 million children between the ages of 11 and 13 were also out of school. The arrival of COVID-19 worsened an already inadequate education system in the region, and such worrying figures require a multi-faceted approach to CCE (UNICEF, 2021b). As discussed earlier, the report on Youth Perspectives on Climate Change and Education in South Asia (UNICEF, 2021a) uncovers the apprehensive and worried ways in which young people think of climate change. The barriers to school enrollment in the region coupled with the fear and anxieties of climate change experienced by young students present a key opportunity for the integration of CCE in curricula.

The national policies of South Asian countries which focus on climate action by targeting public awareness, capacity building, and institutional training may also prove as essential factors in advancing climate change adaptation measures in the region as they would approach CCE in non-formal sectors.

### Recommendations

While implementation of policies is lacking, there are still avenues existing in South Asia to mainstream CCE in educational institutions and local communities. Ministries of climate and environment, different non-governmental organizations, and various programs funded by international and national bodies are working within South Asian countries to impart CCE on young students and the general population at institutional and local levels. These include but are not limited to Pakistan Institute for Environment-Development Action Research (PIEDAR), JAAGO Foundation in Bangladesh, Kathmandu Environmental Education Project (KEEP) in Nepal, Island Development and Environmental Awareness Society (IDEAS) in Maldives, and Bhutan Foundation. Regional bodies are also working collaboratively in the region to build on and improve development practices. The South Asia Co-operative Environment Programme (SACEP), the South Asia Youth Environment Network (SAYEN), and the South Asian Association for Regional Cooperation (SAARC) are good examples to further action on climate change which is both collective and reflective in nature (SAARC, 2015). For CCE to work in South Asia, there must be a reliable collaboration between different governmental and non-governmental bodies, as well as visible fiscal resources provided and safeguarded through policies which can be actionable to achieve the SDG13 goal on climate action.

Additionally, this collaboration must be strengthened with the use of relevant theoretical frameworks and local knowledge systems which support effective strategies for adaptation, preparedness, response, and recovery in the event of a climate-induced challenge. Universities and colleges in South Asia have a moral obligation to step up and develop their curriculum so that their students are prepared to act when faced with a climate-related threat but also to adapt their practices such as agricultural and infrastructural development. As South Asia is a vulnerable region, many countries within it are experiencing astounding levels of socio-economic inequality, and governments must do more to highlight CCE at all levels of education, as well as promote the teaching of sustainable practices in low-income and rural areas of the region.

**Fig. 3:**
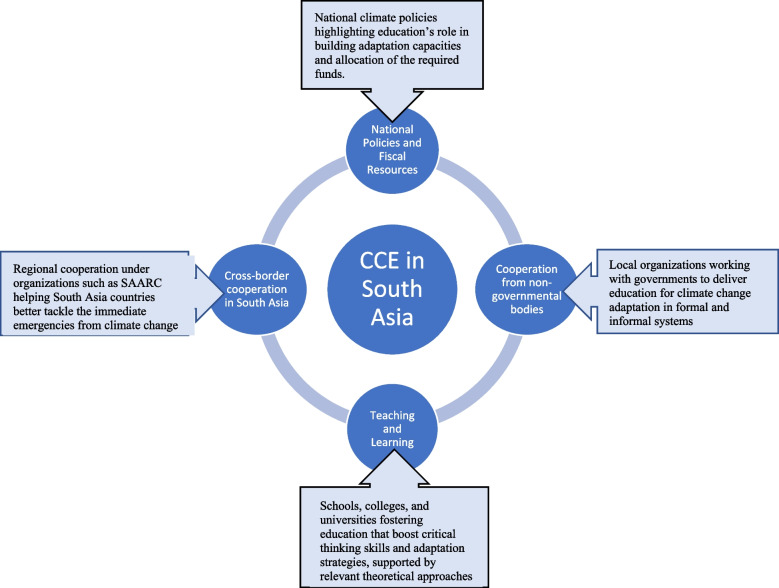
A support framework for a sustainable and impactful education for climate change adaptation in South Asia

As suggested in Fig. 3, effective climate-related educational programs must commence with the crafting of policies that highlight the centrality of education to building the capacity needed to adapt to the adverse effects of climate change. It is also expedient for this to have the necessary fiscal support. Corruption is endemic in many developing countries, including those in South Asia, as such, any fiscal appropriation should be underlined with responsibility, transparency, and accountability. Furthermore, it is not enough to end at the level of policy. Policies must be actionable, and this can be realized once different sectors in South Asia are working together to exchange knowledge on climate change adaptation and devising strategies for implementation, in line with shared objectives. Furthermore, educational programs at schools, colleges, and universities intended to support climate change adaptation must be anchored on critical pedagogy to bolster change in specific localities. In addition, a sustainable network of collaboration needed is not limited between governmental and non-governmental bodies but should also involve cross-border initiatives as countries within the region face similar climate-induced challenges, and shared solidarity and capacity building can be better coordinated and optimized.

## Conclusion

The latest Working Group II Contribution to the Sixth Assessment Report of the Intergovernmental Panel on Climate Change (IPCC) (Shaw et al. 2022) highlights the extent to which South Asian countries will face climate-related challenges. The current climate policies of South Asian countries may be a step in the right direction, but they are wholly inadequate in advancing an impactful CCE that supports climate change adaptation in the region. However, there are evidences to suggest that South Asian countries have clear intentions to boost climate change adaptation knowledge and practices. As asserted, countries such as Sri Lanka and Bhutan have pledged to include essential components of CCE at different levels of their education systems. On the other hand, countries such as India and Bangladesh are focusing more on developing capacity by strengthening their institutions and building knowledge management centers. Nepal and Pakistan are among countries that are strategizing ways to incorporate topics of climate change, environment, and sustainability into their schools. Modes of delivery of CCE captured include workshops and activities in local communities to build climate resilience, and knowledge acquisition in schools and higher institutions regarding conservation and disaster management practices. In the non-formal sectors, education on issues, impacts, and information on ways to adapt to climate change is being provided through public awareness in most of the targeted countries.

While the analysis of policies and practices of CCE that supports adaptation in South Asia presents interesting findings, gaps exist, owing to a number of factors such as the lack of readiness and resources. The need to foster inclusivity via a multi-disciplinary approach and cross-sector, as well as cross-border collaborative initiatives are important factors to be considered. The interplay between varied factors that underpin climate-induced challenges also demands a system thinking approach in tackling them, and this can be imbedded in different disciplines that advance knowledge on climate change adaptation. Furthermore, teaching and learning intended to support climate change adaptation in the region must consider relevant theoretical lenses such as ToC, critical pedagogy, place attachment theory, and praxis, as this can spur a sustainable and impactful response.

A support framework for a sustainable and impactful education for climate change adaptation in South Asia has been advanced, emphasizing key contribution from national policies and fiscal provision; local organizations working with governments to deliver education for climate change adaptation in formal and informal systems; schools, colleges, and universities fostering education that boost critical thinking skills and adaptation strategies, supported by relevant theoretical approaches; and regional cooperation between South Asian countries, exercising solidarity in addressing the immediate and common emergencies from climate change across the region.

It is worth noting that several of the climate-induced challenges captured in this paper are also widespread in other developing countries, beyond the shores of the South Asia region. In this regard, the framework and recommendations generated here can also be implemented in contexts in Africa, and South America, for instance. Future studies on the subject and context can benefit from a more robust and in-depth approach that would capture interviews or focus group discussions with key stakeholders to make more sense of some of the findings articulated, as content analysis of documents may not establish the entire state of affair.
